# Diagnostic Accuracy Improvement of an Updated HEART Score to Predict Coronary Artery Disease as Detected by Coronary Computed Tomography Angiography

**DOI:** 10.3390/jcm15041424

**Published:** 2026-02-11

**Authors:** Michele Della Rocca, Stefano Ferdico, Nicola Cosentino, Alice Bonomi, Andrea Baggiano, Manuela Muratori, Saima Mushtaq, Laura Salvini, Matteo Biroli, Edona Leka, Gianluca Pontone, Marco Grazi, Emilio Assanelli

**Affiliations:** 1 Department of Emergency and Urgent Cardiac Care, Centro Cardiologico Monzino IRCCS, 20138 Milan, Italy; 2 Department of Perioperative Cardiology and Cardiovascular Imaging, Centro Cardiologico Monzino IRCCS, 20138 Milan, Italy; 3 Department of Biostatistics, Centro Cardiologico Monzino IRCCS, 20138 Milan, Italy; 4 Department of Biomedical, Surgical and Dental Sciences, University of Milan, 20122 Milan, Italy

**Keywords:** acute coronary syndrome, HEART score, computed tomography angiography, diagnostic accuracy

## Abstract

**Background:** The HEART score is a widely used risk-stratification tool in suspected acute coronary syndrome (ACS), but it still suffers from several limitations. We aim to assess its diagnostic accuracy for predicting coronary computed tomography angiography (CCTA) findings and explore possible enhancement by integrating additional clinical variables. **Methods:** In this retrospective, observational study, consecutive patients presenting to the Emergency Department with suspected ACS and undergoing CCTA were analyzed. The study assessed the HEART score’s diagnostic accuracy for predicting significant coronary artery stenosis (defined as ≥70% stenosis at CCTA) and explored improvements by integrating additional clinical variables for low-to-moderate-risk patients. **Results:** Three hundred seventy-nine patients were enrolled (age: 61 ± 15 years; male: 57%). According to the HEART score, 27% were at low risk, 67% moderate risk, and 6% high risk, with a prevalence of significant CAD of 7%, 27%, and 67%, respectively. The area under the curve (AUC) of the HEART score to predict significant CAD was 0.68. Male gender (OR = 1.76, 95% CI 1.03–3.02), right bundle branch block (OR = 4.15, 95% CI 1.66–10.40), and hemoglobin (OR = 1.21) and glucose levels (OR = 1.01) independently predicted significant coronary stenosis at CCTA in patients at low-to-moderate risk. Integrating these variables into the HEART score, the AUC improved from 0.68 to 0.74 (*p* = 0.004), with a net reclassification improvement of 13.5% (*p* = 0.032). **Conclusions:** Integrating additional clinical variables into the HEART score improves its accuracy to predict significant coronary artery stenosis at CCTA in suspected ACS patients at low-to-moderate risk. Tailoring assessments with these variables supports more accurate patient management and highlights the potential for more comprehensive diagnostic approaches.

## 1. Introduction

Acute coronary syndrome (ACS) continues to present a critical challenge, representing one of the leading causes of morbidity and mortality worldwide [1]. The timely and accurate diagnosis of ACS is crucial, as it significantly influences patient outcomes and healthcare resource utilization. Emergency departments (EDs) often face the task of rapidly distinguishing ACS from non-cardiac causes of chest pain—a common symptom that brings patients to seek urgent care. The HEART score, an acronym representing History, ECG, Age, Risk factors, and Troponin levels, was developed as a clinical decision-making tool to help stratify risk and guide management protocols for patients presenting with chest pain indicative of possible ACS [2,3]. Despite its widespread validation and clinical adoption, the HEART score faces some criticism. Indeed, while its overall sensitivity for predicting MACE is high, it does not eliminate the risk of adverse cardiovascular outcomes in low-to-moderate-risk patients, with studies reporting event rates of 4.6% for patients at low risk and between 16.6 and 38% for those at moderate risk [3,4,5,6]. This is particularly relevant given that an acceptable risk threshold for ED physicians to discharge a patient without further testing is generally considered to be below 0.5% [7].

Coronary computed tomography angiography (CCTA) has emerged as a powerful modality for evaluating patients with suspected ACS, particularly in those with low-to-moderate risk. By providing detailed anatomical visualization of the coronary arteries, CCTA offers a high negative predictive value for ruling out significant coronary artery disease [8,9,10,11,12]. Consequently, the HEART score may help identify patients more likely to have pathological CCTA findings, aiding in the appropriate selection of candidates for CCTA referral.

We therefore conducted a study to evaluate the diagnostic accuracy of an updated HEART score incorporating additional clinical variables, identified within this study, to predict CCTA findings, as a reference for significant CAD in the ED setting, for patients presenting with suspected ACS.

## 2. Materials and Methods

This is a retrospective, observational, single-center study conducted at Centro Cardiologico Monzino, Milan, Italy. The study was approved by the Institutional Review Board of Centro Cardiologico Monzino (protocol number: NP1076). The study period spans from April 2022 to July 2024 and retrospectively included 379 consecutive patients admitted to our ED for suspected ACS. Only patients whose initial assessments did not allow for definitive exclusion or confirmation of ACS, based on clinician judgment, and referred for urgent coronary CCTA were enrolled. Patients aged < 18 years, those who explicitly opted out of research participation at our institution, and patients with incomplete data required to calculate the HEART score or analyze CCTA results were excluded from the study.

Data were collected retrospectively by reviewing the medical records of eligible patients. Demographic and clinical characteristics, including age, sex, comorbidities, risk factors for cardiovascular disease (e.g., family history of coronary artery disease, diabetes mellitus, arterial hypertension, smoking, dyslipidemia, and chronic kidney disease), and presence of chest pain, were retrieved. The HEART score components were collected in all patients, including clinical history, standard 12-lead ECG findings (normal, non-specific changes, and significant ST-segment deviations), age, traditional risk factors, and serial high-sensitivity troponin I levels.

All CCTA examinations were performed using a Revolution CT scanner (GE Healthcare, Milwaukee, WI, USA), with the following parameters: slice configuration of 256 × 0.625 mm with scintillator detector (Gemstone detector, GE Healthcare, Milwaukee, WI, USA); gantry rotation time of 280 ms; tube voltage of 120 KVp and 100 KVp in patients with BMI >30 kg/m’ and ≤30 kg/m’, respectively; and effective tube current of 500 mA. One-beat axial scan was used in all patients, with variable padding ranging between 70% to 80% and 40% to 80% of the cardiac cycle in patients with HR < 65 beats/min and those with ≥65 beats/min, respectively. All patients received a 70 mL bolus of iodixanol 320 (320 mg/mL, e Visipaque, GE Healthcare) at an infusion rate of 6.2 mL/s, followed by 50 mL of saline solution. All scans were performed using the bolus tracking technique by using visual assessments to determine the timing of image acquisition. The results were interpreted independently by two specialists (a radiologist and a cardiologist), both with dedicated expertise in cardiovascular imaging, to ensure consistency and accuracy of reporting. The findings were considered significant CAD in the presence of at least one ≥70% stenosis in a coronary segment or ≥50% when referring to the left main coronary artery (hereafter referred to as “positive CCTA”).

Continuous variables are presented as mean ± SD and were compared using the t-test for independent samples. Variables not normally distributed are presented as median and interquartile ranges and were compared with the Wilcoxon rank sum test. Categorical data were compared using the chi-square or Fisher’s exact test, as appropriate.

Receiver operating characteristic (ROC) curves were calculated, and the area under the receiver operating characteristic curve (AUC) with a 95% confidence interval (CI) was used to measure the ability of the HEART score to predict the presence of at least one severe coronary artery stenosis, as assessed by CCTA. The ROC curves were calculated in the overall study population and in patients with low-to-moderate risk HEART scores. The independent predictors of severe coronary artery stenosis in patients with low-to-moderate risk HEART scores were assessed by logistic regression, considering the variables found to be statistically different according to CCTA findings (excluding variables already included in the HEART score items). Results are presented as odds ratios (ORs) with 95% CIs. The variables identified as independent predictors of significant coronary stenosis at CCTA were incorporated into the HEART score. The AUCs for the HEART score alone and with the addition of the identified clinical variables in low-to-moderate-risk patients were compared by the DeLong test. Net reclassification improvement was then applied to assess the potential additional predictive value for a positive CCTA when these clinical variables are included with the HEART score in low-to-moderate-risk patients.

All tests were 2-tailed, and *p* < 0.05 was required for statistical significance. All analyses were performed using SAS version 9.4 (SAS Institute). Reclassification statistics were assessed with the SAS macros published by Cook and Ridker.

## 3. Results

A total of 379 patients (mean age 61 ± 15 years, male sex 57%) were included in the study. The baseline clinical characteristics, cardiovascular risk factors, cardiovascular therapy taken before ED presentation, admission ECG, results of major laboratory tests, HEART score, and corresponding risk category, along with the findings from the CCTA, are shown in Tables S1 and S2 (see Supplementary Materials). The baseline characteristics included typical cardiovascular risk factors, such as arterial hypertension (51%), dyslipidemia (38%), and a family history of coronary artery disease (22%). Only a minority of the study patients underwent prior myocardial revascularization, with 1% having undergone PCI and 0.5% CABG. The median high-sensitivity troponin I at presentation was 8 ng/L, with a BNP level of 90 pg/mL. ECG findings at presentation varied, with 33% of patients showing normal ECGs, while others exhibited T-wave inversions (19%), non-specific repolarization abnormalities or left ventricular hypertrophy (25%), and more specific changes such as ST-segment depression (9%). Right bundle branch block was present in 5% of the overall cohort.

**Figure 1:**
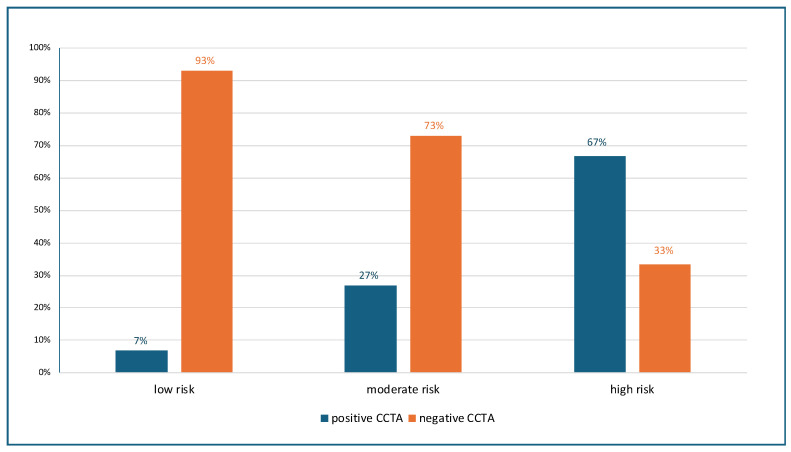
Percentage of negative and positive CCTAs in patients with low, moderate, and high HEART score risk categories. The distribution of patients across HEART score categories was as follows: 27% low risk (*n* = 103), 67% moderate risk (*n* = 252), and 6% high risk (*n* = 24). CCTA—coronary computed tomography angiography.

The HEART score stratified patients into low (27%), moderate (67%), and high risk (6%) categories. Therefore, most patients were categorized as low or moderate risk according to the HEART score. Overall, 91 patients (24%) were found to have significant CAD at CCTA. Figure 1 shows the prevalence of significant CAD in patients at low, moderate, and high risk HEART score categories. Of note, 7% of patients in the low risk category, 27% in the moderate risk category, and 67% in the high risk category were found to have a positive CCTA scan. Considering patients at the low and moderate risk together, 75 patients out of 355 (21%) had a significant stenosis at CCTA. Figure S1 (see Supplementary Materials) shows the AUCs for predicting a positive CCTA in the overall study population [AUC 95% CI 0.71 (0.67–0.78)] (Panel A) and in patients with low-to-moderate risk HEART scores [AUC 95% CI 0.68 (0.61–0.73)] (Panel B).

Based on these findings and considering that most of the study patients were in the low and moderate risk categories, reflecting daily clinical practice, we focused our analysis on these two categories combined. A detailed comparison of low-to-moderate-risk patients with negative versus positive CCTA findings is shown in Table 1. Among the variables found to be significantly different (and excluding those already included in the HEART score), independent predictors of a positive CCTA scan for a significant coronary stenosis in patients with low and moderate HEART scores are reported in Table S3 (see Supplementary Materials). Logistic regression analysis identified male gender (OR = 1.76, 95% CI 1.03–3.02, *p* = 0.0391) and RBBB (OR = 4.15, 95% CI 1.66–10.40, *p* = 0.0023) as significant predictors of positive CCTA results. For each 1 g/dL increase in hemoglobin and for each 10 mg/dL increase in glucose, the risk of having a positive CCTA also significantly increased (OR = 1.21 and OR = 1.07, respectively).

**Figure 2:**
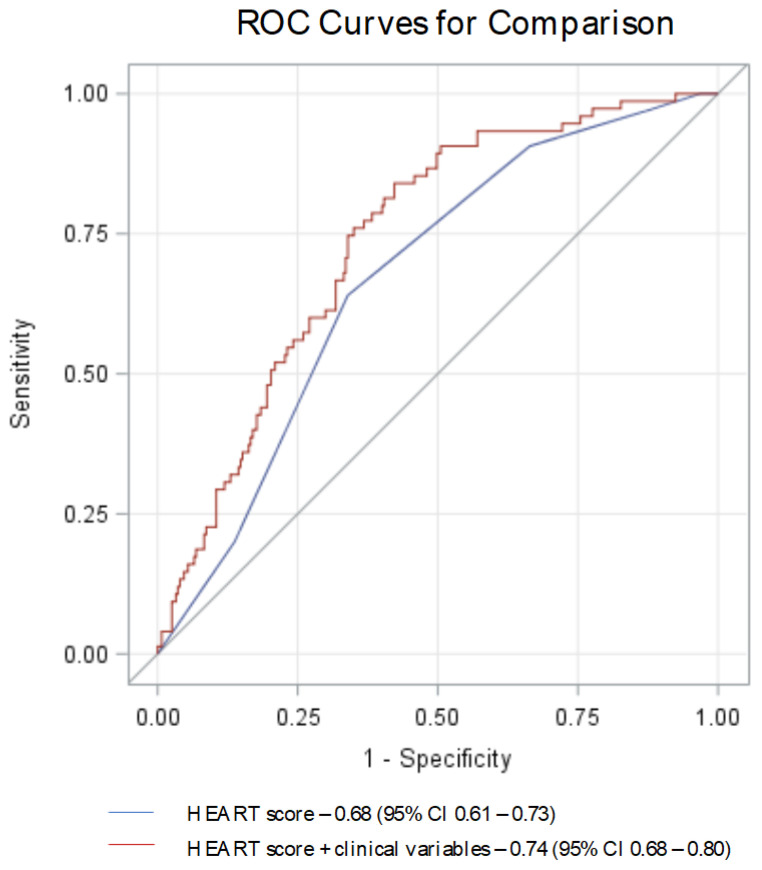
ROC curves for predicting a positive CCTA in patients with low-to-moderate risk HEART scores: AUC with (red) and without (blue) the integration of additional clinical variables (male gender, RBBB, Hb, blood glucose). AUC—area under the curve; CCTA—coronary computed tomography angiography; Hb—hemoglobin; RBBB—right bundle branch block; ROC—receiver operating characteristic.

In patients with low-to-moderate risk HEART score categories, enhancing the HEART score model with these additional variables improved the AUC for predicting a positive CCTA from 0.68 (95% CI 0.61–0.73) to 0.74 (95% CI 0.68–0.80), indicating a significant improvement in diagnostic accuracy (*p* = 0.004, Figure 2).

**Figure 3:**
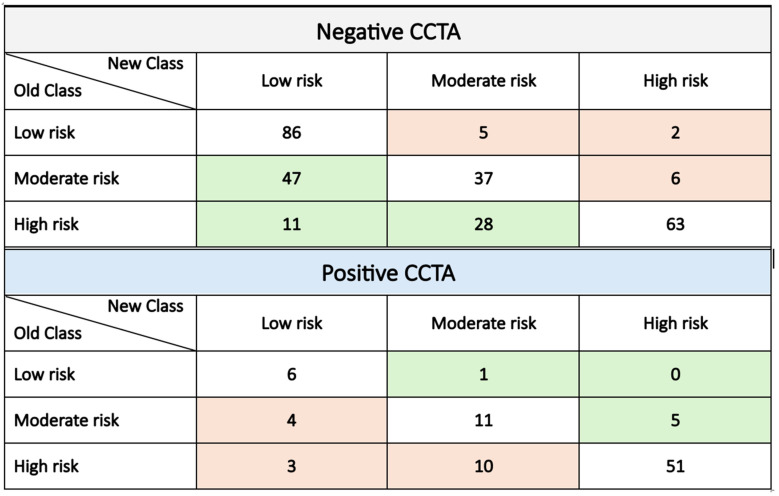
Risk category reclassification with the addition of clinical variables (male gender, RBBB, Hb, and blood glucose) to the HEART score-based model. Green boxes show the number of patients who were appropriately reclassified (i.e., downgraded for patients with a negative CT scan and upgraded for patients with a positive CT scan); conversely, red boxes show patients who were inappropriately reclassified.

According to the net reclassification analysis, the addition of these four variables to the HEART score allowed for the proper classification 13.5% of patients (*p* = 0.032). Figure 3 shows the reclassification of patients between HEART score risk categories for both negative and positive CCTA patients. The enhanced model maintained robust prognostic accuracy across relevant patient subgroups (e.g., according to age, diabetes, gender, hypertension, and hemoglobin), ensuring the reliable prediction of positive CCTA outcomes regardless of clinical and laboratory variations (see Supplementary Materials Figure S2).

## 4. Discussion

The results of this study highlight the potential benefits of integrating additional clinical variables into the HEART score, thereby improving its ability to identify significant coronary artery stenosis, as assessed by CCTA, in patients with suspected ACS. Importantly, in this real-world setting, most of the patients presenting to the ED with a suspected ACS were classified as low-to-moderate risk, with one-fifth of them showing significant coronary stenosis at CCTA. Including variables, such as male gender, RBBB, hemoglobin, and glucose levels, in the HEART score enhanced its predictive accuracy for detecting significant coronary stenosis. These findings address, at least in part, a critical limitation of the traditional HEART score, which, although useful, lacks granularity in identifying higher-risk individuals within the low-to-moderate risk categories. The observed improvement in the AUC and NRI supports the utility of the enhanced model in providing clinicians with a robust tool for decision-making in this clinical setting.

An important limitation of our study should be acknowledged upfront, namely that a “positive CCTA”, defined as the presence of a ≥70% coronary stenosis, does not directly correspond to acute coronary syndrome, the need for revascularization, or short-term clinical outcomes, such as 30-day major adverse cardiac events. Owing to the retrospective and observational nature of the study, as well as the consequent limited statistical power, our primary aim cannot be to propose a definitive diagnostic tool for ACS. Rather, the purpose of this analysis is to explore whether selected clinical and laboratory parameters, integrated with an established risk score, may potentially assist in emergency department risk stratification, helping to distinguish patients who may benefit from hospital admission and further invasive diagnostic evaluation from those who could be safely discharged after an inconclusive initial assessment.

We recognize that the definitive confirmation of the absence of ACS would ideally require systematic follow-up of discharged patients. However, given the retrospective and observational design of our study, our approach relies on the well-established and extensively validated high negative predictive value of coronary CT angiography, which has consistently been shown to safely exclude obstructive coronary artery disease and short-term adverse events in the emergency department setting.

We also acknowledge that coronary artery disease is biologically complex and cannot be fully captured by luminal stenosis severity alone. Contemporary CCTA studies have demonstrated that coronary calcium burden, plaque composition and vulnerability, and perivascular inflammatory status provide complementary prognostic and pathophysiological information beyond traditional clinical scores, highlighting the multifaceted nature of CAD risk. In particular, recent evidence has linked coronary calcification with plaque phenotype and perivascular inflammation as key contributors to disease activity and instability [13]. Although these advanced imaging markers were not available in our dataset and could therefore not be incorporated into the present model, they represent an important direction for future research. Within this evolving imaging-based paradigm, our findings should be interpreted as an initial attempt to refine clinical decision tools by integrating readily available clinical parameters, thereby positioning the enhanced HEART score within a broader framework of anatomical and biological risk assessment.

Acute coronary syndrome remains a major global health challenge, with timely and accurate diagnosis being crucial for patient outcomes. Traditional reliance on clinical history, ECG findings, and cardiac biomarkers, while effective, often falls short in low-to-moderate-risk patients. In the ED, where rapid decision-making is essential, the HEART score has been widely adopted due to its simplicity and effectiveness in stratifying patients based on their risk of major adverse cardiovascular events [3,5]. However, its limitations become apparent in its inability to fully mitigate the risk of adverse cardiovascular outcomes in low- or moderate-risk patients, indicating a significant unmet need in current diagnostic strategies [14,15]. Indeed, while high risk HEART score patients are straightforwardly managed through hospitalization and monitoring, low-to-moderate-risk patients often face the possibility of being discharged home. Notably, also patients who are classified as low risk and are deemed safe for discharge from the ED may still encounter significant coronary events, highlighting a gap in current diagnostic capabilities [5,6].

In this clinical scenario, CCTA has emerged as a highly effective method for evaluating suspected ACS, especially in low-to-moderate-risk patients. Despite the existence of multiple definitions of “critical” stenosis and the growing interest in plaque-based and biologically driven CCTA classifications, we consider the ≥70% stenosis threshold to be the most appropriate and clinically useful cutoff in this specific emergency department setting, as it is widely accepted to indicate severe obstructive disease with potential implications for invasive diagnostic strategies and patient disposition. Indeed, a coronary stenosis ≥ 70% detected on CCTA is strongly correlated with the risk of major cardiovascular events (MACEs) according to the international literature. Data from major trials, such as PROMISE and SCOT-HEART, show that the presence of significant stenosis (≥70%) at CCTA identifies a population at increased risk of myocardial infarction and cardiovascular death compared to patients with non-obstructive or absent disease [16,17,18,19].

In the SCOT-HEART trial, the presence of obstructive stenosis (≥50%, but the risk increases further with stenosis ≥ 70%) was associated with an almost double risk of coronary death or non-fatal infarction compared to patients without significant stenosis (hazard ratio ~2), and the concomitant presence of high-risk plaque characteristics further increased the risk (hazard ratio up to 11.5 compared to normal coronary). The meta-analysis of Bamberg et al. confirms that the presence of significant stenosis at CCTA is an independent predictor of cardiovascular events, with a hazard ratio greater than 6 for stenosis ≥ 50% and annualized event rates around 12% [19].

The PROMISE trial showed that patients with stenosis ≥70% at CCTA have similar event rates to those with positive functional tests, while most events occur in the presence of obstructive disease, but a non-negligible share also occurs in the presence of non-obstructive disease [17]. However, stenosis ≥70% remains the most related anatomical cut-off with major events and with the clinical decision of revascularization [16,17].

Two recent studies explored the HEART score’s role in managing patients with suspected ACS in the ED, with a special focus on its association with significant coronary stenosis at CCTA. Han et al. demonstrated that the HEART score is a good predictor of both ACS and significant coronary artery stenosis, with a marginal better performance for significant coronary artery stenosis (AUC of 0.73 vs. 0.71 for ACS) [20]. Arslan et al. showed that integrating the HEART score with CCTA enhances diagnostic efficiency and that reserving CCTA for intermediate-risk patients (HEART score 3–6) reduces unnecessary imaging by 22% while maintaining high sensitivity (97.8%) and negative predictive value (99.6%) for 30-day major adverse cardiac events [21]. Thus, both studies highlight the HEART score’s value in optimizing ED workflows in patients with suspected ACS. However, neither study assessed whether incorporating additional simple and easily obtained clinical variables could enhance the HEART score’s ability to predict significant coronary artery stenosis.

In our study, 7% of low-risk and a significant 27% of moderate-risk patients were found to have severe coronary stenosis, as assessed by CCTA. Our findings align with existing research that challenges the HEART score’s sensitivity, particularly in identifying patients at higher risk among those considered at low-to-moderate risk [3,5,22,23]. We also reported that incorporating additional variables such as male gender, RBBB, hemoglobin, and glucose levels significantly enhances the HEART score’s capacity to predict significant coronary stenosis within these risk categories. The enhanced model, which showed increased AUC (from 0.68 to 0.74) and NRI (+13.5%), reflects a growing trend towards more comprehensive and personalized diagnostic approaches that utilize a wider range of clinical and laboratory data, moving beyond traditional scoring systems.

The mechanisms underlying the association between the additional clinical variables identified in this study and the presence of severe coronary stenosis, as assessed by CCTA, cannot be definitely inferred from the data alone. Nevertheless, several hypotheses can be proposed based on existing physiological and clinical knowledge. Firstly, the male gender is inherently linked to a higher prevalence of coronary artery disease due to biological and hormonal differences, which may predispose males to a more significant cardiovascular risk [22]. This heightened risk could potentially explain the observed association, as males may be more likely to present with severe stenosis even when categorized into lower risk groups by traditional scoring systems. The presence of RBBB on an ECG might signal underlying conduction abnormalities that could be a surrogate marker for latent cardiac pathology. RBBB could potentially indicate a predisposition to structural heart changes or ischemic conditions that are not captured by the standard HEART score, thus correlating with severe stenosis [24]. The association observed between hemoglobin levels and significant CAD should be interpreted with caution, as hemoglobin is likely to reflect a constellation of clinical and demographic factors—such as hydration status, smoking-related disease, sex, and chronic comorbidities—rather than a direct pathophysiological link to atherosclerotic burden. However, one possible hypothesis is that elevated hemoglobin levels might contribute to increased blood viscosity and greater coronary vascular resistance, which could reduce coronary blood flow and increase myocardial workload, thereby potentially increasing the propensity for thrombosis and resultant coronary stenosis [4,25,26]. Finally, high glucose levels may derive from an inflammatory and adrenergic response to potential ongoing ischemic stress and/or may reflect unknown diabetes mellitus. Moreover, high glycemic values are known to disrupt ischemic preconditioning, promote apoptosis, reduce nitric oxide bioavailability, impair endothelial function, enhance platelet aggregation, and stimulate coagulation [27]. Interestingly, Alavi-Moghaddam et al. demonstrated that integrating admission blood glucose levels into the HEART score improved diagnostic performance in patients with suspected ACS, with an increase in the AUC in ROC analysis for major adverse cardiac events [28]. These hypotheses offer potential explanatory models to better understand the observed correlations within the study, and though speculative, they align with current understandings in ACS pathophysiology. However, further research is needed to elucidate the exact causal pathways and validate these associations in diverse clinical settings. Accordingly, our results should be regarded as hypothesis-generating and require confirmation in larger and more diverse populations. Prospective studies across different clinical settings will be needed to validate these associations and to clarify whether the identified variables may meaningfully contribute to CAD risk stratification.

The enhanced HEART score model may have relevant clinical implications, particularly for low-to-moderate-risk patients who might otherwise be discharged without further investigation. In emergency settings, efficient and accurate triage is paramount to prevent adverse cardiac events while optimizing resource utilization. The enhanced score allows for the improved identification of patients who may benefit from further diagnostic evaluation, such as CCTA, or more aggressive therapeutic interventions, thus preventing potentially adverse outcomes associated with undetected coronary artery disease.

This study has some strengths and limitations. The study’s strengths lie in its comprehensive analysis of a well-characterized patient cohort and the innovative approach to enhancing the HEART score with a special focus on CCTA. Indeed, all patients included in this analysis presented at the ED with suspected ACS and underwent CCTA. However, its retrospective design constrains causal inference and may introduce selection biases inherent in data extraction from medical records. Our study population was selected after inconclusive first-line assessments; while this process inevitably depends on operator judgment and may introduce relevant selection bias, it reflects real-world clinical decision-making in the ED and likely explains the relatively high overall prevalence of CAD observed in our cohort (24%). It should be noted that a model derived under these conditions may not apply to EDs with different CCTA thresholds, scanners, pre-test probabilities, or hs-troponin pathways. An additional limitation of our study is the absence of an external validation cohort; all analyses were conducted exclusively on the derivation cohort of patients who underwent coronary computed tomography angiography (CCTA) based on clinical judgment. Furthermore, the single-center nature of the study could limit the applicability of the findings to other populations due to demographic or institutional variations. The cohort’s specific characteristics, such as the low proportion of patients undergoing prior revascularization, may not fully represent broader clinical settings, potentially affecting the generalizability of the enhanced model. To build on these findings, future research should aim to validate the enhanced HEART score in prospective, multicentric studies encompassing diverse populations to assess its generalizability. It would also be beneficial to explore the incorporation of other potential biomarkers and genetic factors that could further refine risk stratification processes. Investigating the integration of machine learning algorithms with the HEART score variables could provide insights into developing dynamic and adaptive risk models that evolve with patient data trends.

## 5. Conclusions

In conclusion, this study demonstrated the potential of an enhanced HEART score model to improve the accuracy for predicting significant coronary artery stenosis in patients presenting to the ED with suspected ACS and considered to be at low-to-moderate risk. By incorporating additional simple clinical and laboratory variables, the model offers a promising tool for risk stratification, addressing critical gaps in current diagnostic practices. These findings underscore the need for ongoing research to refine diagnostic frameworks and improve patient care in the context of ACS.

## Figures and Tables

**Table 1:** Clinical differences in low-to-moderate-risk patients, considered together with negative and positive CCTA.

	Negative CCTA (*n* = 280)	Positive CCTA (*n* = 75)	*p*-Value
Age (years)	59 ± 15	64 ± 11	0.009
Male gender	153 (54%)	51 (68%)	0.03
BMI	25.6 ± 4.7	26.6 ± 4.9	0.19
**History**			
Smoking	51 (18%)	19 (25%)	0.16
Hypertension	120 (42%)	52 (69%)	<0.0001
Diabetes mellitus	17 (6%)	11 (15%)	0.014
Dyslipidemia	94 (34%)	31 (41%)	0.21
Family history of CAD	53 (19%)	25 (33%)	0.008
Obesity	20 (7%)	11 (15%)	0.04
CKD	2 (1%)	1 (1%)	0.60
AF/AFL	34 (13%)	7 (9%)	0.42
Obstructive CAD	4 (1.6%)	5 (7%)	0.31
Prior ischemic stroke	6 (2%)	1 (1%)	0.65
COPD	7 (3%)	3 (4%)	0.48
Prior PTE/DVT	0	2 (3%)	0.006
**Presentation parameters**			
Systolic blood pressure (mmHg)	136 ± 22	140 ± 20	0.20
Diastolic blood pressure (mmHg)	81 ± 12	84 ± 12	0.04
Heart rate (bpm)	77 ± 23	80 ± 19	0.36
SpO_2_ (%)	98 ± 1.4	98 ± 1.5	0.24
**Presentation ECG**			
Normal	99 (35%)	26 (35%)	0.91
AF/AFL	18 (6%)	4 (5%)	0.72
ST-segment depression	22 (8%)	4 (5%)	0.46
T-wave inversion	56 (20%)	10 (13%)	0.19
Nonspecific repolarization abnormalities	62 (22%)	16 (21%)	0.88
Left ventricular hypertrophy	9 (3%)	1 (1%)	0.38
LBBB	13 (5%)	1 (1%)	0.19
RBBB	10 (3%)	10 (13%)	0.001
**Blood tests**			
Hemoglobin (g/dL)	13.9 ± 1.5	14.4 ± 1.5	0.027
Creatinine (mg/dL)	0.92 ± 0.2	0.93 ± 0.2	0.51
Blood glucose (mg/dL)	111.8 ± 36	122.3 ± 42.2	0.032
C-reactive protein (mg/L)	2.5 (1–6)	3 (1–8)	0.74
Hs-TnI at presentation (ng/L)	6.3 (2.6–36)	9.4 (4.9–37.5)	0.79
BNP (pg/mL)	84 (29–220)	60 (12–329)	0.64

AF/AFL—atrial fibrillation/atrial flutter; ACS—acute coronary syndrome; BNP—brain natriuretic peptide; CAD—coronary artery disease; CABG—coronary artery bypass graft; CKD—chronic kidney disease; COPD—chronic obstructive pulmonary disease; DVT—deep vein thrombosis; Hs-TnI—high-sensitivity troponin I; LBBB—left bundle branch block; PCI—percutaneous coronary intervention; PTE—pulmonary thromboembolism; RBBB—right bundle branch block.

## Data Availability

The data supporting the findings of this study are available from the corresponding author upon reasonable request.

## Supplementary Materials

The following supporting information can be downloaded at: https://www.mdpi.com/article/10.3390/jcm15041424/s1, Table S1: Baseline characteristics of the overall study population; Table S2: Presentation parameters of the overall study population; Table S3: Odds ratios and 95% confidence intervals of independent predictors of a significant coronary stenosis at CCTA in patients at low-to-moderate risk HEART score; Figure S1: ROC curve for predicting a positive CCTA in the overall study population (Panel A) and in patients with low-to-moderate risk HEART score (Panel B); Figure S2: Accuracy (AUC values) for predicting a positive CCTA of the HEART score-based model with the addition of clinical variables (male gender, RBBB, Hb, and blood glucose) stratified according to most relevant patients’ subgroups.

## Abbreviations

The following abbreviations are used in this manuscript:

ACS	Acute coronary syndrome
AF/AFL	Atrial fibrillation/atrial flutter
AUC	Area under the curve
BMI	Body mass index
BNP	Brain natriuretic peptide
CABG	Coronary artery bypass graft
CAD	Coronary artery disease
CI	Confidence interval
CKD	Chronic kidney disease
COPD	Chronic obstructive pulmonary disease
CCTA	Coronary computed tomography angiography
ED	Emergency Department
ECG	Electrocardiogram
Hb	Hemoglobin
HR	Heart rate
Hs-TnI	High-sensitivity troponin I
KVp	Kilovoltage peak
LBBB	Left bundle branch block
MACE	Major adverse cardiovascular event
NPV	Negative predictive value
OR	Odds ratio
PCI	Percutaneous coronary intervention
PTE	Pulmonary thromboembolism
RBBB	Right bundle branch block
SD	Standard deviation
