# Supplementary material for: Diagnostic Accuracy Improvement of an Updated HEART Score to Predict Coronary Artery Disease as Detected by Coronary Computed Tomography Angiography
Source: J Clin Med. 2026 Feb 11;15(4):1424. doi: 10.3390/jcm15041424 (PMC12942576; doi:10.3390/jcm15041424)
Supplement: Supplementary file 1 [file jcm-15-01424-s001.zip › jcm-4120499-supplementary.pdf]

Supplemental Digital Content

Table S1. Baseline characteristics of the overall study population.

Baseline demographic characteristics, cardiovascular risk factors, comorbidities, and medications of interest of the overall study population included in the analysis (n = 379). Continuous variables are reported as mean ± standard deviation, and categorical variables as number (percentage).

	Overall study population (n=379)
Age (years)	61±15
Male gender	217 (57%)
BMI	26 ±5
History	
Smoking	78 (21%)
Hypertension	192 (51%)
Diabetes mellitus	34 (9%)
Dyslipidemia	142 (38%)
Family history of CAD	82 (22%)
Obesity	31 (8%)
Chronic kidney disease	3 (1%)
Atrial fibrillation	44 (12%)
Obstructive CAD	9 (2,5%)
Prior Ischemic stroke	8 (2%)
COPD	10 (3%)
Prior PTE/DVT	2 (0,5%)
Medications of interest	
ACEi/ARB	147 (39%)
Beta blockers	86 (23%)
Calcium channel blockers	57 (15%)
Lipid lowering drugs	108 (28%)
Antiplatelet drugs	84 (22%)
Oral anticoagulant drugs	36 (9%)
Antihyperglycemic drugs	28 (7%)

Abbreviations: ACEi=angiotensin-converting enzyme inhibitors; ARB=angiotensin receptor blockers; CAD=coronary artery disease; COPD=chronic obstructive pulmonary disease; DVT= deep vein thrombosis; PTE= pulmonary thromboembolism.

Table S2. Presentation parameters of the overall study population.

Clinical presentation parameters at emergency department admission, including vital signs, electrocardiographic findings, laboratory values, HEART score risk categories, and coronary computed tomography angiography (CCTA) results in the overall study population (n = 379). Continuous variables are reported as mean ± standard deviation or median (interquartile range), as appropriate, and categorical variables as number (percentage).

	Overall study population (n=379)
**Presentation parameters**	
Systolic blood pressure(mmHg)	138±23
Dyastolic blood pressure (mmHg)	82±12
Heart rate (bpm)	77±22
SpO2(%)	98±2
**Presentation ECG**	
Normal	125 (33%)
AF/AFL	24 (6%)
ST-segment depression	34 (9%)
T-wave inversion	72 (19%)
Nonspecific repolarization abnormalities/LVH	96 (25%)
LBBB	17 (4%)
RBBB	21 (5%)
**Blood tests**	
Hemoglobin(g/dL)	14±1,5
Creatinine(mg/dL)	0.93±0,2
Blood glucose (mg/dL)	115±40
C-reactive protein(mg/L)	2,4 (1-6,3)
Hs-TnI at presentation(ng/L)	8 (3-41)
BNP (pg/mL)	90 (29-233)
**HEART score**	
Low risk	101 (27%)
Moderate risk	252 (67%)
High risk	24 (6%)
**CCTA**	
Non severe stenosis	288 (76%)
Severe stenosis	91 (24%)

Abbreviations: AF/AFL=atrial fibrillation/atrial flutter; BNP= brain natriuretic peptide; CCTA = coronary computed tomography angiography; Hs-TnI= high-sensitivity troponin I; LBBB = left bundle branch block; RBBB = right bundle branch block.

Table S3. Odds ratios and 95% confidence intervals of independent predictors of a significant coronary stenosis at CCTA in patients at low-to-moderate risk HEART score.

Multivariable logistic regression analysis identifying independent clinical predictors of significant coronary artery stenosis on coronary computed tomography angiography (CCTA) among patients classified as low to moderate risk by the HEART score. Odds ratios (ORs) and 95% confidence intervals (CIs) are reported. Effect estimates for hemoglobin are expressed per 1 g/dL increase, and effect estimates for blood glucose are expressed per 10 mg/dL increase.

	OR (95% CI)	P-value
Male gender	1.76 (1.03-3.02)	0.0391
RBBB	4.15 (1.66-10.40)	0.0023
Hemoglobin (g/dL)	1.21 (1.02-1.44)	0.0278
Blood glucose (mg/dL)	1.07 (1.01-1.13)	0.0406

Abbreviations: RBBB = right bundle branch block.

Figure S1. ROC curve for predicting a positive CCTA in the overall study population (Panel A) and in patients with low-to-moderate risk HEART score (Panel B).

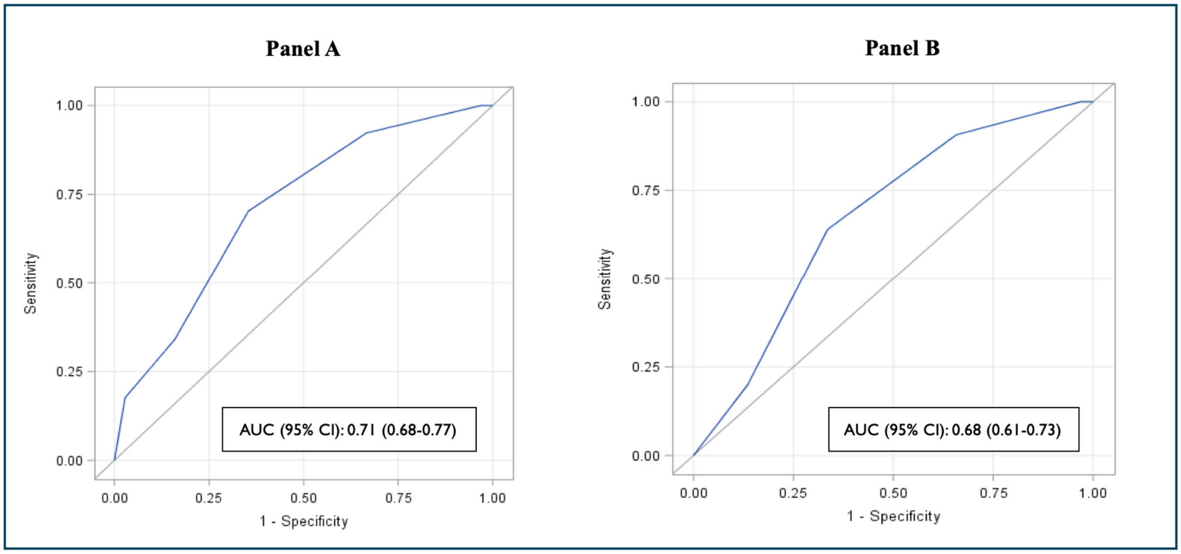

CCTA = coronary computed tomography angiography.

Figure S2. Accuracy (AUC values) for predicting a positive CCTA of the HEART score-based model with the addition of clinical variables (male gender, RBBB, Hb, and blood glucose) stratified according to most relevant patients' subgroups.

The figure shows that the enhanced model maintains a good and consistent discriminative performance across multiple patient subgroups, including age, sex, cardiovascular risk factors, ECG findings, biomarker status, and chest pain characteristics, supporting the robustness of the model in heterogeneous patient subsets.

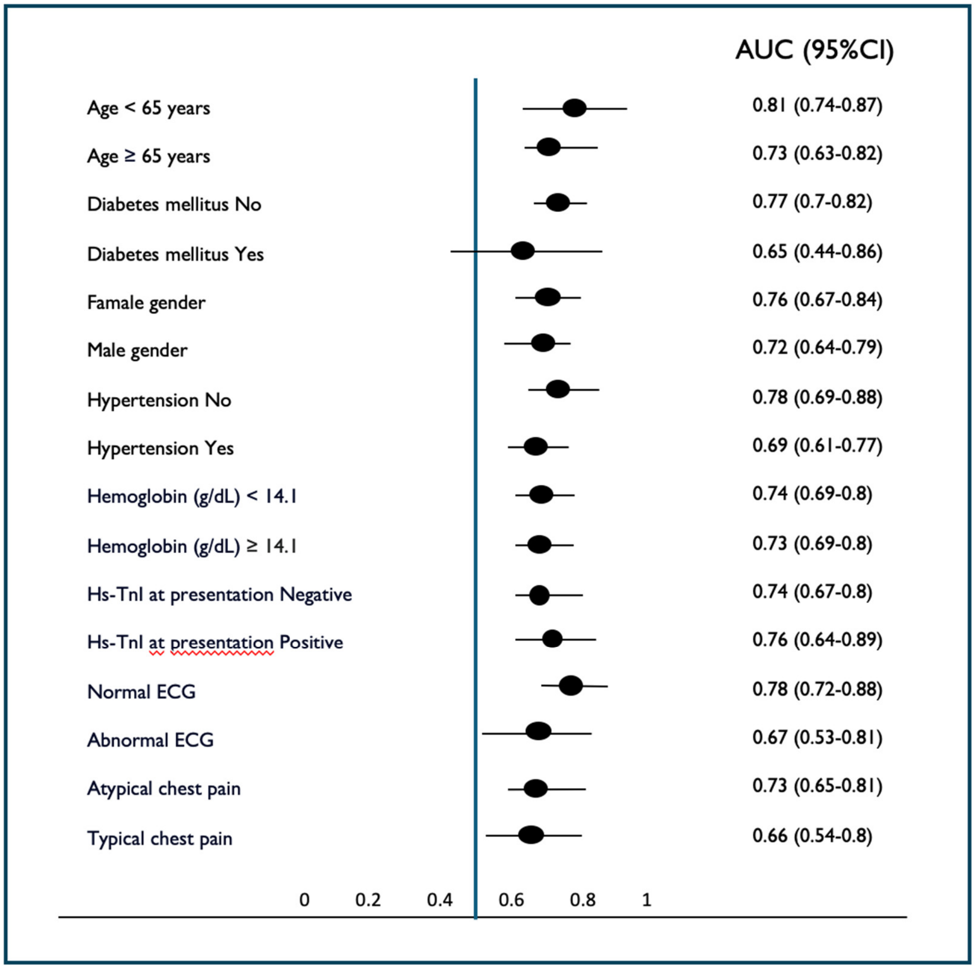

AUC = area under the curve; CCTA = coronary computed tomography angiography; Hb = hemoglobin; RBBB = right bundle branch block.
